# Correlations among disability, anti-AQP4 antibody status and prognosis in the spinal cord involved patients with NMOSD

**DOI:** 10.1186/s12883-021-02171-2

**Published:** 2021-04-09

**Authors:** Jung Lung Hsu, Ming-Feng Liao, Kuo-Hsuan Chang, Mei-Yun Cheng, Long-Sun Ro

**Affiliations:** 1 Department of Neurology, New Taipei Municipal TuCheng Hospital, Chang Gung Memorial Hospital and Chang Gung University, New Taipei City, Taiwan; 2 Department of Neurology, Chang Gung Memorial Hospital Linkou Medical Center and College of Medicine, Chang-Gung University, Linkou, Taoyuan Taiwan; 3 Graduate Institute of Mind, Brain, & Consciousness, Taipei Medical University, Taipei, Taiwan; 4 Brain & Consciousness Research Center, Shuang Ho Hospital, New Taipei City, Taiwan; 5 Institute of Molecular Medicine, National Tsing Hua University, Hsinchu, Taiwan

**Keywords:** NMOSD, EDSS, Anti-AQP4, MRI

## Abstract

**Background:**

Neuromyelitis optica spectrum disorder (NMOSD) is a rare neuroinflammatory disorder of the central nervous system that typically involves the optic nerve, the spinal cord and other specific brain regions. In relapse of the disease, factors associated with clinical features and lesion severity are important for clinicians to predict disease-related disability.

**Methods:**

We retrospectively analyzed 22 female patients with NMOSD who had spinal cord lesions. Detailed clinical features, onset symptoms, motor disability, relapse episodes, serum aquaporin-4 (AQP4) and myelin oligodendrocyte glycoprotein (MOG) autoantibodies and MRI characteristics were documented to correlate their associations with the nadir and three-month Expanded Disability Status Scale (EDSS) scores. Patients with three-month EDSS scores below four (< 4) were categorized as the good outcome group, while those with scores of four or more (> 4) were categorized as the poor outcome group.

**Results:**

In patients with NMOSD, the mean age was 44.5 ± 12.8 years, and the mean three-month EDSS score was 4.3 ± 1.9. A significantly higher all-limb muscle power score was found in the good EDSS group than in the poor EDSS group (*p* = 0.01). A tendency toward longer follow-up periods and lower anti-AQP4 antibody levels was found in the good outcome group. Serum anti-AQP4 antibodies were present in 86% of patients with NMOSD, and MOG autoantibodies were found in one anti-AQP4 antibody-negative patient (33.3%). In patients with NMOSD, more than 40% of spinal cord lesions were distributed at the middle cervical and upper thoracic levels.

**Conclusions:**

Our findings suggest that EDSS scores and MRC scores at the nadir had significant associations with three-month EDSS scores. The topographic distributions of the spinal cord lesions might relate to different serum anti-AQP4 antibody status. However, further studies will be needed to corroborate this finding.

## Introduction

Neuromyelitis optica spectrum disorder (NMOSD) is a group of chronic inflammatory and demyelinating disorders that are characterized by optic neuritis, transverse myelitis and extensive brain lesions in locations such as the brainstem, the area postrema and the diencephalic regions [1, 2]. After the discovery of highly specific serum immunoglobulin G antibodies that target the water channel protein aquaporin-4 (AQP4-IgG), this serological marker was incorporated into the revised NMOSD diagnostic criteria and became the standard for clinical and research purposes [1]. NMOSD is different from multiple sclerosis (MS) in that the former causes greater disability due to severe optic nerve damage and longitudinally extensive transverse myelitis (LETM), fewer brain magnetic resonance imaging (MRI) lesions and the presence of anti-AQP4 antibody in the serum and cerebrospinal fluid (CSF) [3, 4]. From a pathophysiological perspective, NMOSD is an autoimmune water channelopathy that predominantly affects astrocytes in the central nervous system (resulting in secondary demyelination) [5], while MS is a heterogeneous, multifactorial, immune-​mediated disease that is caused by complex gene–environment interactions [6]. The prevalence of NMOSD in various studies ranges from 0.5 to 4 per 100,000 individuals, and the annual incidence is < 1/million individuals; therefore, it is categorized as a rare disease [7–9]. In Taiwan, the prevalence of NMOSD is unknown, but it affects a significantly higher proportion of middle-aged female than male patients, exhibits a high relapse rate, and results in greater functional disability than conventional multiple sclerosis [10].

The variability of disease severity in NMOSD has been documented; some patients have a disease course with frequent relapses and early motor disability, while others may have only a single attack without accumulating significant relapse-associated disability despite many years of disease [11]. The reasons for this heterogeneity are not clear and make it difficult to predict future outcomes. Recently, several studies, including one on a large international dataset, showed confounding factors for disease severity that included ethnic differences, onset age, sex, initial onset symptoms, recurrent episodes and serological features [12–14]. In the current work, we explored the relationships connecting clinical features to disease severity and relapse episodes after the diagnosis of NMOSD.

## Materials and methods

### Study design and patient population

We retrospectively analyzed the initial clinical presentations, neurological examinations, MRI features and serum anti-AQP4 antibody profiles of a total of 22 NMOSD patients with spinal cord lesions from 2002 to 2018. The study protocol was approved by the institutional review board of the Chang Gung Memorial Hospital, Linkou, Taoyuan, Taiwan (IRB number: 201800769B0). All methods were performed in accordance with the relevant guidelines and regulations. We recorded demographic characteristics; medical histories; and information on the clinical presentations, including the temporal profiles, initial symptoms and neurological examination results. The relevant MRI features, including the topography of the lesions at the initial assessment, were analyzed. The diagnosis of NMOSD was based on the international consensus criteria for that disorder [1]. Our study is a retrospective chart review analysis covering the period from 2002 to 2018. Before the 2015 international consensus criteria for NMOSD were available, NMOSD was diagnosed according to older criteria [15, 16]. Patient(s) with NMOSD might initially present with non-spinal cord symptoms but later develop spinal cord involvement, at which time the patient(s) were recruited for this study. Most of our patients (16 out of 22) were diagnosed with NMOSD after 2008. When patients were anti-AQP4-IgG positive, we used the 2015 international consensus criteria for NMOSD to recruit these patients.

The temporal profiles from the initial onset of symptoms to the nadir of neurological dysfunction were measured in days. The nadir was defined as the point of the worst neurological function, before any improvement or plateau, based on history and neurological examinations. The initially presented symptoms were categorized as optic neuritis symptoms or spinal cord symptoms. The severity of motor disability was measured at the nadir of the disease and three months after symptom onset. For each limb, a Medical Research Council (MRC) score (from 0 to 5) was assigned to represent the muscle power in that limb. The total muscle power in all four limbs was calculated by summating the MRC scores of all the limbs. We also determined the Kurtzke Expanded Disability Status Scale (EDSS) scores at nadir and at three months in patients with NMOSD [17]. According to the treatment data of our study cohort, 20 of 22 patients received various doses of prednisolone, and two patients did not receive any medication. Among the patients receiving prednisolone treatment, four also used azathioprine, two used interferon beta, two used mycophenolate mofetil (MMF) and one used cyclosporine. Serum anti-AQP4 antibody testing was performed by enzyme-linked immunosorbent assay (ELISA) (normal value < 3 units/mL) [18]. Anti-myelin oligodendrocyte glycoprotein (anti-MOG) antibody analysis was carried out in anti-AQP4 antibody-negative patients using a cell-based assay (CBA) with live transfected cells [19]. Patients with EDSS scores < 4 at three months were defined as the good outcome group, while those with EDSS scores > 4 were classified as the poor outcome group. Each patient’s follow-up period (from symptom onset to the date of the last visit) and the number of relapse episodes within that period were also recorded.

### Evaluation of MRI parameters

MRI of the spine obtained within two weeks of admission was reviewed by a board-certified neuroradiologist, who recorded the MR parameters. All studies included axial and sagittal T1- and T2-weighted sequences that imaged the spine and brain. The main characteristic of MRI features in patients with NMOSD is LETM [20]. In order to study LETM, the length of the spinal cord lesion was measured as the sagittal extent of T2 hyperintensity using the number of vertebral body spans. The location of each lesion was recorded according to the vertebral body level (e.g., cervical, thoracic or lumbar). Gadolinium [Gd] enhancement on T1-weighted images was recorded as present or absent. The distribution of lesions in the cross-sections of the spinal cord was classified according to the anterior, posterior, lateral and central regions [21]. The presence of absence of “bright spotty lesions”, defined as the typical NMOSD feature of “very hyperintense spotty lesions on the axial T2-weighted images that are visually more hyperintense than those of surrounding CSF without flow void effects”, was recorded in all patients [22].

### Statistical analyses

All statistical analyses were performed using SPSS (version 21.0; IBM, New York, USA). Continuous variables are expressed as the means ± standard deviations. Categorical variables are presented as counts and ratios. Independent t-tests were performed to compare the mean ages of the groups. Chi-square tests, Fisher’s exact tests and Mann-Whitney U tests were used to compare patients with NMOSD in terms of sex, clinical presentations, three-month outcomes and imaging characteristics. Logistic regression analysis was used to study the associations among clinical symptoms, short-term outcomes and MRI characteristics after adjustments for age and sex effects. Statistical significance was defined as *p* <  0.05.

## Results

**Fig. 1:**
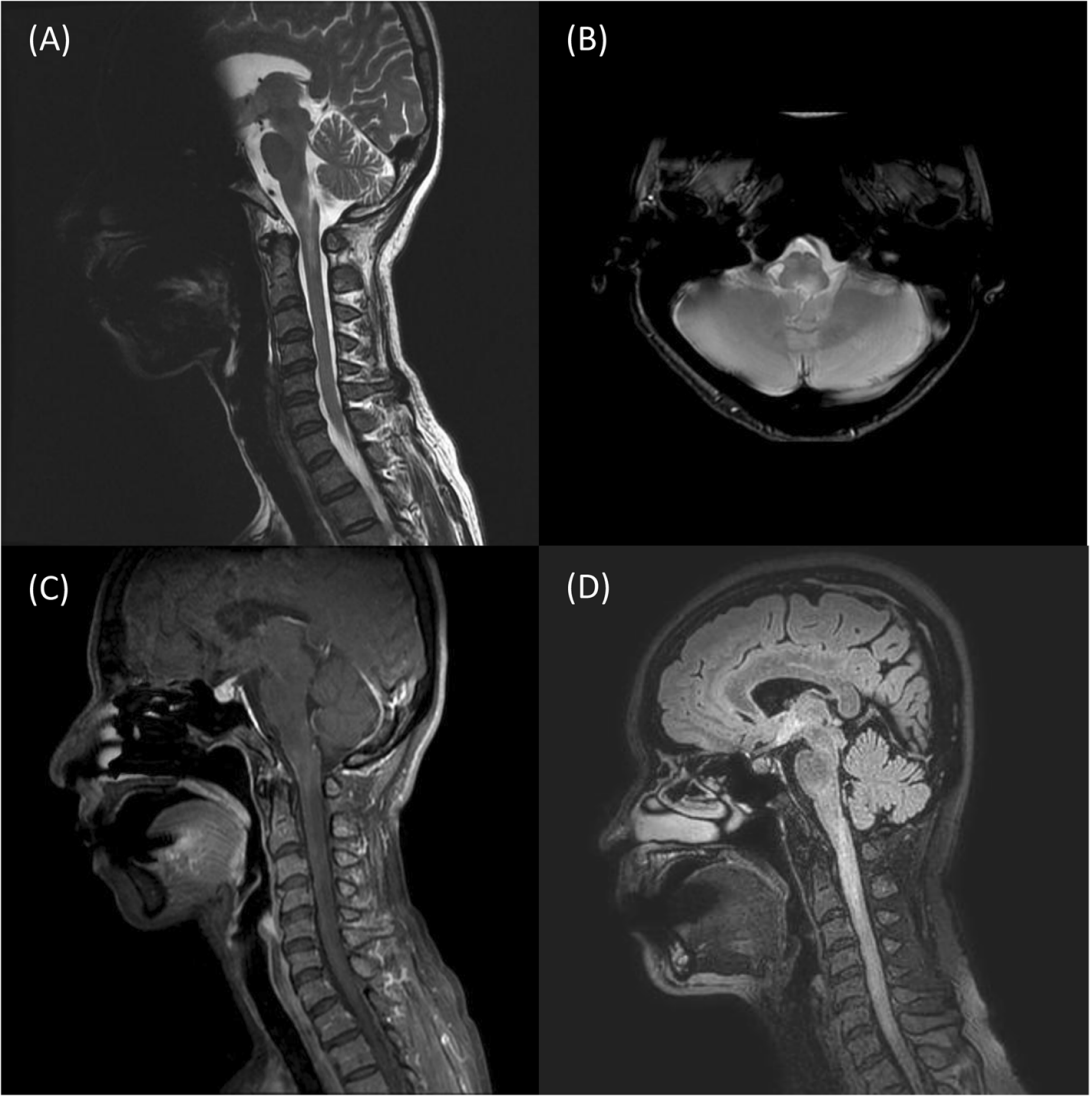
Brain MRI showed a representative case of a patient with NMOSD. **a**. A T2-weighted hyperintensity lesion at the area postrema that extended to the first cervical vertebral body level on the MRI sagittal view. **b**. The brain MRI axial view showed a hyperintense lesion at the area postrema that was more severe on the left side of the medulla. **c**. A [Gd]-enhanced lesion from the medulla to the first cervical vertebral body level on the sagittal-view T1-weighted contrast image. **d**. A hyperintense lesion from the area postrema extended to the secondary vertebral body level on the sagittal-view FLAIR image was noted 2 years after the first attack

Figure 1 shows a representative case of NMOSD. A 47-year-old female had subacute onset of a spinning sensation, unsteady gait and recurrent nausea and vomiting for one week. Sagittal-view brain MRI showed a hyperintense lesion in the area postrema that extended to the first vertebral body (Figs. 1 a & b). Gd-enhanced T1-weighted images showed contrast enhancement in the corresponding regions (Fig. 1 c). Her level of anti-AQP4 antibody was 4.46 units/mL. Her EDSS score at three months was 2. Two years after initial presentation, she had a recurrent attack presenting as bilateral upper-limb numbness for two weeks along with right visual impairment. Brain MRI showed an extended lesion from the area postrema to the secondary vertebral body on the Gd-enhanced T1-weighted images (Fig. 1 d). The patient’s EDSS score at three months was 3.5.

**Fig. 2:**
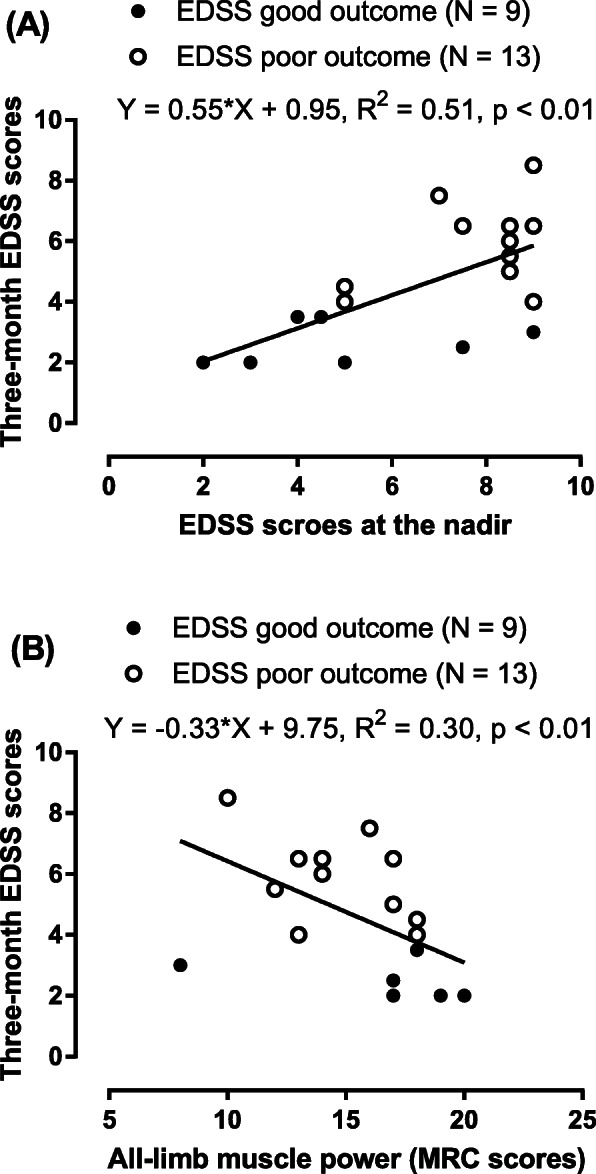
Three-month EDSS scores associated with EDSS scores at the nadir and all-limb MRC scores. **a**: There was a significantly positive association between EDSS at the nadir and three-month EDSS scores. **b**: There was a significantly negative correlation between the all-limb muscle power scores and the three-month EDSS scores in patients with NMOSD

In total, 22 female patients with NMOSD were enrolled in the current study; Table 1 summarizes the features of the good and poor outcome groups. In terms of clinical parameters, there were no significant differences in age, onset-to-nadir time, initial symptoms, follow-up period or number of relapses between the good and poor outcome groups. Significant differences in lower-limb and all-limb muscle power (MRC scores) were found between the good and poor outcome groups (*p* = 0.01 and p = 0.01, respectively). Three months after symptom onset, all-limb, upper-limb and lower-limb muscle power (MRC scores) showed significant differences between the good and poor outcome groups (*p* <  0.01, *p* = 0.02 and p <  0.01, respectively). In longitudinal follow-ups, a longer follow-up period and fewer relapse episodes were found in the good outcome group than in the poor outcome group, although the differences were not significant. Serum anti-AQP4 antibodies were positive in 86% of our patients with NMOSD. Three patients with NMOSD tested negative for anti-AQP4 antibodies. Serum AQP4-IgG levels were lower in the good outcome group than in the poor outcome group (*p* = 0.27). Furthermore, the anti-AQP4 antibody status did not show a significant difference between the good and poor outcome groups (*p* = 0.33). Regression analysis showed a significant positive association between EDSS scores at the nadir and three-month EDSS scores even after adjusting for onset age and the length of spinal cord lesions (R squared = 0.55, *p* <  0.01; Fig. 2a). Regarding MRC scores, regression analysis showed a significant negative association between all-limb muscle power scores at the nadir and three-month EDSS scores (R squared = 0.30, *p* <  0.01; Fig. 2b). All-limb, upper-limb and lower-limb muscle power scores at three months showed significant negative associations with EDSS scores even after adjusting for onset age and the length of spinal cord lesions (all p <  0.01).

**Table 1:** Comparisons of demographic factors, clinical parameters, anti-AQP4 antibody status and MRI features between the good and poor three-month EDSS outcome groups

Group	Good outcome (EDSS < 4, *N* = 9)	Poor outcome (EDSS > 4, *N* = 13)	*p*-values
Onset age (years)	43.1 ± 17.6	42.6 ± 9.1	0.74
Onset to nadir time (days)	7.9 ± 6.5	8.7 ± 6.1	0.71
Initial symptoms			0.68
Optic neuritis	2	2	
Spinal cord lesions	7	11	
All-limb muscle power (MRC score)	17.4 ± 3.7	14.7 ± 2.5	0.01
Upper-limb muscle power (MRC score)	4.8 ± 0.4	4.5 ± 0.6	0.10
Lower-limb muscle power (MRC score)	3.9 ± 1.6	2.9 ± 1.1	0.01
EDSS scores at the nadir	4.3 ± 2.5	7.9 ± 1.4	< 0.01
Three-month all-limb muscle power (MRC scores)	19.1 ± 0.9	16.2 ± 2.6	< 0.01
Three-month upper-limb muscle power (MRC scores)	4.9 ± 0.2	4.5 ± 0.5	0.02
Three-month lower-limb muscle power (MRC scores)	4.6 ± 0.4	3.6 ± 1.2	< 0.01
Three-month EDSS scores	2.5 ± 0.7	5.8 ± 1.3	< 0.01
Sphincter incontinence (Y:N)	2:7	6:7	0.25
Follow-up period (Months)	108.1 ± 25.5	74.4 ± 58.6	0.12
Relapse episodes	4.6 ± 2.6	5.7 ± 5.9	0.97
Anti-AQP4 antibody level (Unit/mL)	67.4 ± 81.4	125.1 ± 98.2	0.27
Anti-AQP4 antibody status (Negative: Positive)	2:7	1:12	0.33
MRI features			
The length of spinal cord lesions (vertebral body span)	4.9 ± 1.7	4.9 ± 1.4	0.84
Axial anterior pattern (Y:N)	1:8	2:11	0.77
Axial central pattern (Y:N)	5:4	8:5	0.78
Axial lateral pattern (Y:N)	5:4	6:7	0.66
Axial posterior pattern (Y:N)	3:6	7:6	0.34
Bright spotty lesions (Y:N)	5:4	9:4	0.51
Gd enhancement (Y:N)	7:2	7:6	0.64

*EDSS* Expanded Disability Status Scale, *AQP4* aquaporin-4, *MRC* Medical Research Council, *Gd* Gadolinium, *Y* yes, *N* No

Regarding initial symptoms, four patients with NMOSD had optic neuritis and spinal cord lesions, and 18 patients had only spinal cord lesions. Although the group with only spinal cord lesions had higher three-month EDSS scores than the group with optic neuritis, there was no significant difference between the two groups (EDSS score: 4.7 ± 2.1 vs. 3.4 ± 1.1, *p* = 0.25). In terms of relapse episodes, the group with only spinal cord lesions had more relapse episodes than the group with optic neuritis, but the difference was not significant (relapse episodes: 5.4 ± 5.3 vs. 3.0 ± 1.1, *p* = 0.30).

**Fig. 3:**
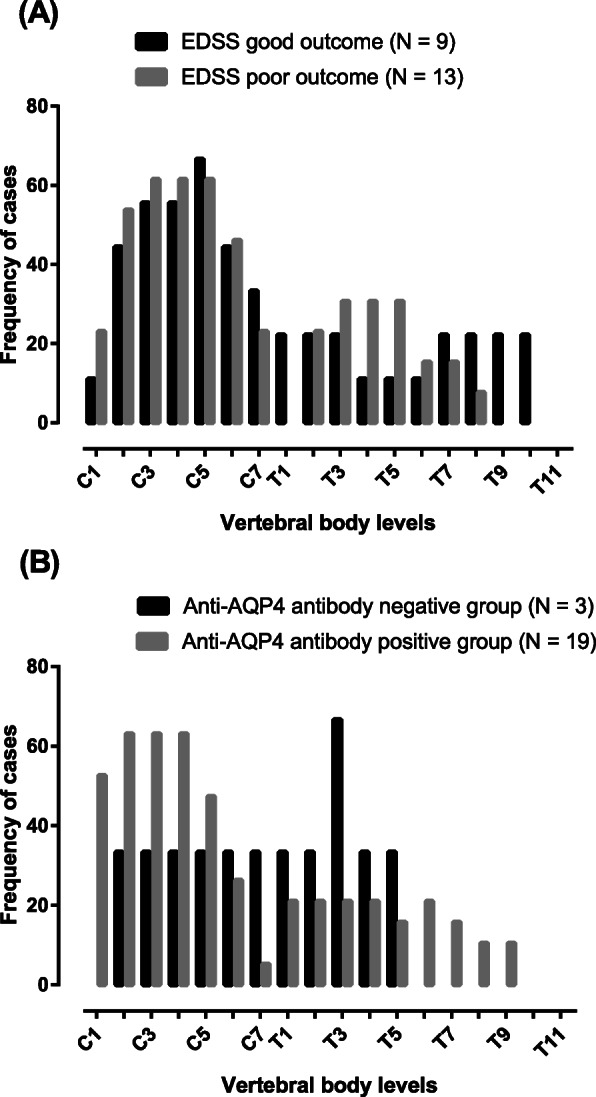
Topographical distributions of the spinal cord lesions in patients with NMOSD according to EDSS classification and anti-AQP4 antibody status. **a**. Most of the spinal cord lesions were located at the middle cervical levels. The poor outcome group had more T3-T5 vertebral body lesions than the good outcome group; the groups were defined by three-month EDSS scores (> 4: poor outcome group; < 4: good outcome group). **b**. The serum anti-AQP4 antibody-positive group had more C1-C5 vertebral body lesions than the serum anti-AQP4 antibody-negative group, while the serum anti-AQP4 antibody-negative group had more upper thoracic vertebral body lesions than the serum anti-AQP4 antibody-positive group

In terms of MRI features, Fig. 3a shows the topographic distribution of LETM. In both EDSS outcome groups, more than 40% of the lesions were located at the C3 to C6 vertebral body levels. The second most common lesion location was at the T3 to T5 vertebral body levels. Spinal cord lesion length (defined as the number of vertebral bodies spanned) was not significantly different between the good and poor EDSS outcome groups (*p* = 0.84; Table 1). We plotted the topographic distribution of LETM according to positive and negative serum anti-AQP4 antibody results, and we found that the serum anti-AQP4 antibody-positive group had more frequent lesions at the cervical levels (C1-C5), while the anti-AQP4 antibody-negative group had more frequent lesions at the upper thoracic levels (Fig. 3b). The axial pattern of MRI lesions, bright spotty lesions and Gd enhancement in MRI did not show significant differences by EDSS score classification or serum anti-AQP4 antibody status classification.

## Discussion

In the current study, we retrospectively analyzed clinical characteristics, anti-AQP4 antibody levels, imaging features and EDSS scores in 22 patients with NMOSD. We found that three-month EDSS scores showed significant associations with EDSS scores and all-limb muscle power scores at the nadir but not with onset age, length of LETM or serum anti-AQP4 antibody levels. Patients with NMOSD who had positive serum anti-AQP4 antibody results were more likely to have upper to middle cervical lesions, while NMOSD patients with negative serum AQP4 antibody results were more likely to have upper thoracic lesions.

### Three-month EDSS scores and clinical features

Our recent prospective observational study showed that the 4-year conversion rate from first-episode idiopathic inflammatory demyelinating disease to MS was 14.5%, while the conversion rate to NMOSD during the same interval was 3.3%; these rates were lower than those in Western countries [23]. However, patients with NMOSD exhibited many relapses (1.0/year) and displayed higher EDSS scores than conventional MS patients in our country [10]. Prediction of disease prognosis at an early point is important because it can assist physicians in planning future treatments and making decision jointly. In addition, early implementation of different treatment strategies and immunotherapies for different subgroups of NMOSD may be required for the prevention of future relapses. Kitley et al. and other researchers showed that ethnicity, sex, onset age and attack phenotype may correlate with relapse rates and clinical outcomes [12, 14]. Based on previous studies [15, 24], MRI and cerebrospinal fluid variables cannot predict the clinical course or severity of NMSD, but clinical features can. In addition, several studies showed that anti-AQP4 antibody status had no apparent association with EDSS scores in patients with NMOSD [25–27]. In the current study, significant differences in lower-limb MRC scores but not upper-limb MRC scores were found between the good and poor EDSS outcome groups (*p* = 0.01 and *p* = 0.10, respectively). In addition, the lower-limb and all-limb muscle power scores showed a significantly negative association with three-month EDSS scores; however, onset age, initial symptoms, serum anti-AQP4 antibody status and length of spinal cord lesions on MRI were not significantly associated with three-month EDSS scores in this study. EDSS scores were associated with lower-limb muscle power scores and served as a parameter of ambulation at high scores. Before a patient reaches an EDSS score of 4, upper extremity function is assessed mainly based on pyramidal, cerebellar, and sensory functional systems [28]. These results indicated that the associations between limb muscle power scores and EDSS scores were mainly based on lower-limb function.

### Characteristics of spinal cord lesions in patients with NMOSD

Regarding the topographic distribution of LETM, most of the lesions were at the cervical levels (C3-C6), and the second most common lesion location was at the upper thoracic levels (T2-T5). Lesions predominantly involving the cervicomedullary junction are a common feature in anti-AQP4 antibody-related NMOSD [29]. These locations were different from those of vascular insult to the spinal cord (spinal cord infarction), in which lesions were more frequent in the lower thoracic and lumbar regions [21]. Several reports have shown that acute spinal cord infarction may mimic myelitis [30, 31]. From an anatomical point of view, the middle cervical cord receives its blood supply from radicular arteries fed by the extracranial vertebral artery, while the upper thoracic cord receives its blood supply from radicular arteries fed by the aorta [32, 33]. We speculated that in these areas, the spinal cord contains abundant collateral circulation that is at risk for immune-related pathology [34]. One pathological study has shown that anti-AQP4 antibody immunoreactivity and humoral immunity are consistently lost from the early stage of lesion development in NMOSD, notably in the perivascular regions with complement and immunoglobulin deposition [35]. These features of NMOSD are distinct from those of vascular infarction as well as those of normal controls [36].

### Serum anti-AQP4 antibody status associated with the length of spinal cord lesions

In our study, 86% of patients with NMOSD showed positive results for serum anti-AQP4 antibody levels. Previous studies showed anti-AQP4 antibody positivity ratios ranging from 41 to 80% [10, 37, 38]. Serum anti-AQP4 antibody status has associations with disease activity, progression and relapse episodes in patients with NMOSD [39, 40]. It has also been demonstrated by MRI that the status and concentration of serum anti-AQP4 antibodies are associated with the length of spinal cord lesions and the severity of motor disability [37]. Our study showed a tendency toward increased spinal cord lesion length on MRI in the serum anti-AQP4 antibody-positive group (*p* = 0.14). However, there were no significant differences in all-limb muscle power scores or relapse episodes between the serum anti-AQP4-positive and anti-AQP4-negative groups. These results might be attributed to the small sample size and heterogeneity of seronegative NMOSD patients in our study.

### Limitations

Several limitations of our study should be addressed. First, only a small number of patients were recruited for the study. Our cohort included females only, and we also divided the entire cohort to establish a subgroup of NMOSD patients presenting mainly with spinal cord lesions. Thus, because of selection bias, our results cannot be generalized to all NMOSD patients. Second, we used an ELISA rather than a CBA to measure anti-AQP4 antibody status. The former assay has lower sensitivity (60%) than the latter (68%), which is likely to compromise the accuracy with which antibody levels are measured for the correlation with motor disability [41]. In a large cohort study, the false-positive rate of serum anti-AQP4 measurement by ELISA was 0.5%, which was higher than the false-positive rate of CBAs (0.1%) [42–44]. Thus, our findings should be interpreted with caution. In addition, there were only a few NMOSD patients with negative serum anti-AQP4 antibody results. From the literature, approximately 40% of patients with anti-AQP4-negative NMOSD have positive results for antibodies against myelin oligodendrocyte glycoprotein (MOG-IgG) [45]. We performed a MOG-IgG study in our anti-AQP4-negative patients, but only one in three patients (33.3%) showed a positive result, which could not be used for comparisons because of the small number. A previous study showed that seronegative NMOSD patients exhibited distinct features compared with seropositive patients, such as a lack of female preponderance, frequent simultaneous involvement of the bilateral optic nerves, a lower annual relapse rate, and fewer spinal cord lesions [46]. In our study, we did not observe similar findings, possibly because there were too few patients in this group. Third, NMOSD patients in our study received different treatments, which might be a confounding factor for the relapse rate. Different immunotherapies may affect future relapse rates [47]. The time lapse between the onset of symptoms and the initiation of treatment may be another confounding factor [48]. In our study, most of the patients were treated with various doses of prednisolone with or without various immunosuppressants. A long-term prospective study to analyze the effects of medication on relapse episodes in a large sample of NMOSD patients is warranted.

## Conclusions

In patients with NMOSD, EDSS scores and MRC scores at the nadir were significantly associated with three-month EDSS scores. The serum anti-AQP4-positive group had a tendency toward a greater LETM than the serum anti-AQP4 antibody-negative group. Further studies will be needed to verify this finding due to the small sample size and the heterogeneity of seronegative patients in our study.

## Data Availability

Additional clinical data are available from laboratory studies. Please contact LSR at cgrols@adm.cgmh.org.tw if this information is of interest.
